# In situ thermally reduced graphene oxide/epoxy composites: thermal and mechanical properties

**DOI:** 10.1007/s13204-016-0518-y

**Published:** 2016-01-30

**Authors:** Ganiu B. Olowojoba, Salvador Eslava, Eduardo S. Gutierrez, Anthony J. Kinloch, Cecilia Mattevi, Victoria G. Rocha, Ambrose C. Taylor

**Affiliations:** 1 Mechanics of Materials Division, Department of Mechanical Engineering, Imperial College London, London, UK; 2 Centre for Advanced Structural Ceramics, Department of Materials, Imperial College London, London, UK; 3 Department of Chemical Engineering, University of Bath, Bath, UK

**Keywords:** Thermal reduction, Graphene oxide, Reduced graphene oxide, Tensile modulus, Thermal conductivity, Glass transition temperature, Dispersion, In situ processing

## Abstract

Graphene has excellent mechanical, thermal, optical and electrical properties and this has made it a prime target for use as a filler material in the development of multifunctional polymeric composites. However, several challenges need to be overcome to take full advantage of the aforementioned properties of graphene. These include achieving good dispersion and interfacial properties between the graphene filler and the polymeric matrix. In the present work, we report the thermal and mechanical properties of reduced graphene oxide/epoxy composites prepared via a facile, scalable and commercially viable method. Electron micrographs of the composites demonstrate that the reduced graphene oxide (rGO) is well dispersed throughout the composite. Although no improvements in glass transition temperature, tensile strength and thermal stability in air of the composites were observed, good improvements in thermal conductivity (about 36 %), tensile and storage moduli (more than 13 %) were recorded with the addition of 2 wt% of rGO.

## Introduction

Due to its remarkable mechanical, electrical and thermal properties, graphene has evoked a lot of interest amongst researchers working on the development of multi-functional composites for a variety of applications (Potts et al. 2011; Geim and Novoselov 2007; Balandin et al. 2008; Yu et al. 2007; Liang et al. 2009; Kim and Macosko 2009). However, the inert nature of graphene makes it difficult to take full advantage of its excellent properties when used as a filler material in polymeric composites. Such composites are usually characterized by poor filler dispersion and poor interfacial properties between the filler and the polymeric matrix material, arising from the poor compatibility between graphene and many polymeric matrices. Functionalization of the graphene has been widely adopted as one of the ways of overcoming the aforementioned challenges, either by allowing the easy exfoliation of graphite platelets or increasing the compatibility between graphene sheets and the chosen polymeric matrix (Kuilla et al. 2010; Pei and Cheng 2012). Oxidized graphene sheets, known as graphene oxide (GO) sheets, are typically prepared by oxidation and exfoliation of graphite (Hummers and Offeman 1958; Marcano et al. 2010) and have been used as the starting material in the production of graphene-based composites. Although some researchers have reduced GO in situ using hazardous chemical reagents such as hydrazine hydrate (Yousefi et al. 2013), others prefer to reduce GO prior to its incorporation in composites (Liang et al. 2009; Potts et al. 2011).

In the present work, reduced graphene oxide (rGO)/epoxy composites are produced in a facile, scalable and commercially viable way. This involves dispersing freeze-dried GO in a diglycidyl ether of bisphenol-A (DGEBA) epoxy resin by shear mixing. The GO is then reduced in situ (to rGO) using a relatively high-temperature cure schedule for the epoxy resin system, after adding the hardener. The thermal and mechanical properties of the composites produced are characterized and the viability of the processing technique is demonstrated. The advantages of this processing route when compared to those adopted by others are: (1) it does not involve the use of solvents or hazardous GO reducing agents such as hydrazine hydrate; and (2) in situ reduction of the GO during the curing of the epoxy eliminates the extra step in the production process of having to reduce the GO prior to incorporation in the matrix.

## Experimental

### Materials

A standard DGEBA resin (Araldite LY556; Huntsman, UK), having an epoxide equivalent weight (EEW) of 185 g/eq, was used. The curing agent was an accelerated methylhexahydrophthalic acid anhydride (Albidur HE600; Evonik, Germany), having an anhydride equivalent weight (AEW) of 170 g/eq.

### Graphene oxide synthesis

A suspension of graphene oxide was prepared via a modified Tour et al. (Marcano et al. 2010) synthesis in a custom-built rig, using natural graphite flakes (150–500 µm, Sigma-Aldrich). This was freeze-dried using a Powerdry LL1500 freeze dryer (Thermo Scientific, UK).

### Composite preparation

The freeze-dried GO, having an average lateral size of 33 ± 17 µm, was dispersed in the DGEBA epoxy resin using a three-roll mill (80E; Exakt, Germany) as described elsewhere (Olowojoba et al. 2013). A stoichiometric amount of the anhydride (Albidur HE600) was then added to the GO/DGEBA mixture (i.e. the ratio of DGEBA to anhydride was kept at 185:170, according to their equivalent weights). The resulting mixture was stirred at 500 rpm for 15 min at a temperature of 60 °C under atmospheric pressure using an overhead stirrer. The mixture was degassed at −1000 mbar and 60 °C for 15 min to remove trapped air bubbles. The degassed mixture was poured into preheated rectangular steel moulds with internal dimensions of 150 × 80 × 3 mm^3^ and cured at 90 °C for 1 h, and 160 °C for 2 h. A post-cure step of 200 °C for 30 min was carried out to ensure sufficient in situ thermal reduction of the GO, to rGO, in the composite (Tang et al. 2012).

### Thermogravimetric analysis

Thermogravimetric analysis (TGA) was carried out on the composites and GO samples using a TGA/DSC 1 apparatus (Mettler Toledo, UK). A sample of the freeze-dried GO was thermally reduced according to the cure schedule employed for processing the composites (i.e. 90 °C for 1 h; 160 °C for 2 h and 200 °C for 30 min). To investigate the thermally induced dissociation of the GO and rGO, samples were analysed in a nitrogen atmosphere using a heating rate of 5 °C/min. Thermal oxidation of the composites, on the other hand, was analysed in air using a heating rate of 10 °C/min. All samples were analysed over the temperature range 30–800 °C.

### Dynamic mechanical thermal analysis

The thermo-mechanical properties of the rGO/epoxy composites were determined using a Q800 dynamic mechanical thermal analyser (DMTA) from TA Instruments, UK. Rectangular samples with dimensions 60 × 10 × 3 mm^3^ were subjected to temperature sweeps from 30 to 200 °C at a heating rate of 2 °C/min in a dual-cantilever mode using a frequency of 1 Hz and an oscillation strain of 0.05 %. The number average molecular weight, *M*_nc_, between cross-links was calculated from Eq. (1):

1 $$M_{\text{nc}} = \frac{q\rho RT}{{E_{\text{r}} }}$$

where *E*_r_ is the rubbery storage modulus determined at the temperature, *T*, of 453 K (180 °C), *R* is the universal gas constant (8.314 cm^3^ MPa/K mol) and *ρ* is the density of the epoxy determined at room temperature [1.2 g/cm^3^ (Chen et al. 2013)]. Since the density of the epoxy was determined at room temperature, Pearson and Yee (1989) suggest a front factor, *q*, of 0.725.

### X-ray photoelectron spectroscopy

X-ray photoelectron spectroscopy (XPS) analyses were performed using a Theta Probe spectrometer (ThermoFisher Scientific; UK). The instrument was operated at a base pressure of 1 × 10^−9^ mbar. XPS spectra were acquired using an MXR1 monochromated Al Kα X-ray source (*hυ* = 1486.6 eV). An X-ray spot of ~400 μm radius was employed. High-resolution, core level C1s spectra were acquired using a pass energy of 20 eV. The GO sample spectra were charge referenced against the C1s peak at 284.4 eV to correct for charging effects during acquisition.

### Tensile test

Uniaxial tensile tests were conducted on the rGO/epoxy composites at room temperature using a universal tensile testing machine (5584; Instron, UK). Dumbbell specimens having a gauge length of 25 mm were used. A displacement rate of 1 mm/min was adopted. The tensile properties were averaged from the results obtained from a minimum of five specimens. The strain was measured using a clip-on extensometer.

### Thermal conductivity measurement

The thermal conductivities of the rGO/epoxy composites were determined from their thermal diffusivities measured by the laser-flash technique using an LFA-427 Nanoflash (Netzsch, Germany). Three shots each were made on 10 × 10 mm^2^ samples which were 2 mm thick. A temperature range of 30–60 °C was used, at 10 °C intervals, with a laser voltage of 450 V and a pulse width of 0.8 ms. The samples were coated with a thin layer of graphite prior to testing to increase absorption and transmission of the infrared radiation energy.

### Scanning electron microscopy

The morphology of the fracture surfaces of the composites was examined using a Leo 1525 (Carl Zeiss, Germany) scanning electron microscope equipped with a field emission gun (FEG-SEM). An accelerating voltage of 5 kV was used, with the samples being sputter-coated with a thin layer of chromium prior to examination.

## Results and discussion

### Reduction of graphene oxide

**Fig. 1:**
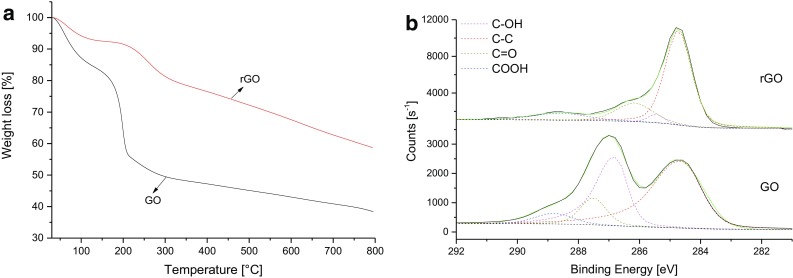
**a** Thermograms of GO and rGO in a nitrogen atmosphere with a heating rate of 5 °C/min; **b** XPS of GO before and after reduction showing a decrease in the concentrations of functional groups present after reduction of GO to rGO

Figure 1a shows the thermograms of the pristine GO and of the rGO reduced according to the temperature schedule adopted for curing the composites. In both thermograms, the weight loss at 100 °C, of 15 % in the case of GO and 7 % in the case of rGO, may be attributed to the evaporation of adsorbed water. A further 30 % weight loss at 200 °C can be observed in the case of GO. This has been attributed to the thermal decomposition of oxygen-containing functional groups (OCFGs) on the surface of the GO. It has previously been shown that most of the OCFGs, which are attached to the basal aromatic plane of a GO sheet (i.e. hydroxyl and epoxy groups), can be removed by annealing at 200 °C (Tang et al. 2012; Jeong et al. 2009), thus partially restoring the thermal, electrical and optical properties of graphene. The OCFGs which are attached to the edges of the GO sheets are however more thermally stable and will only decompose at temperatures higher than 200 °C, as illustrated by the weight loss between 200 and 800 °C. (Pei and Cheng 2012).

Figure 1b shows the C1s XPS spectra of GO and rGO, as well as the associated four-component fit which quantitatively represents the bonds between the carbon atoms on the GO and other moieties. A significant reduction in the OCFGs bonded to carbon atoms can be observed upon reduction of the GO to rGO, in agreement with the TGA results discussed above. These techniques clearly demonstrate the effectiveness of the GO reduction strategy adopted in this work for the preparation of the rGO/epoxy composites.

### Morphologies of the rGO/epoxy composites

**Fig. 2:**
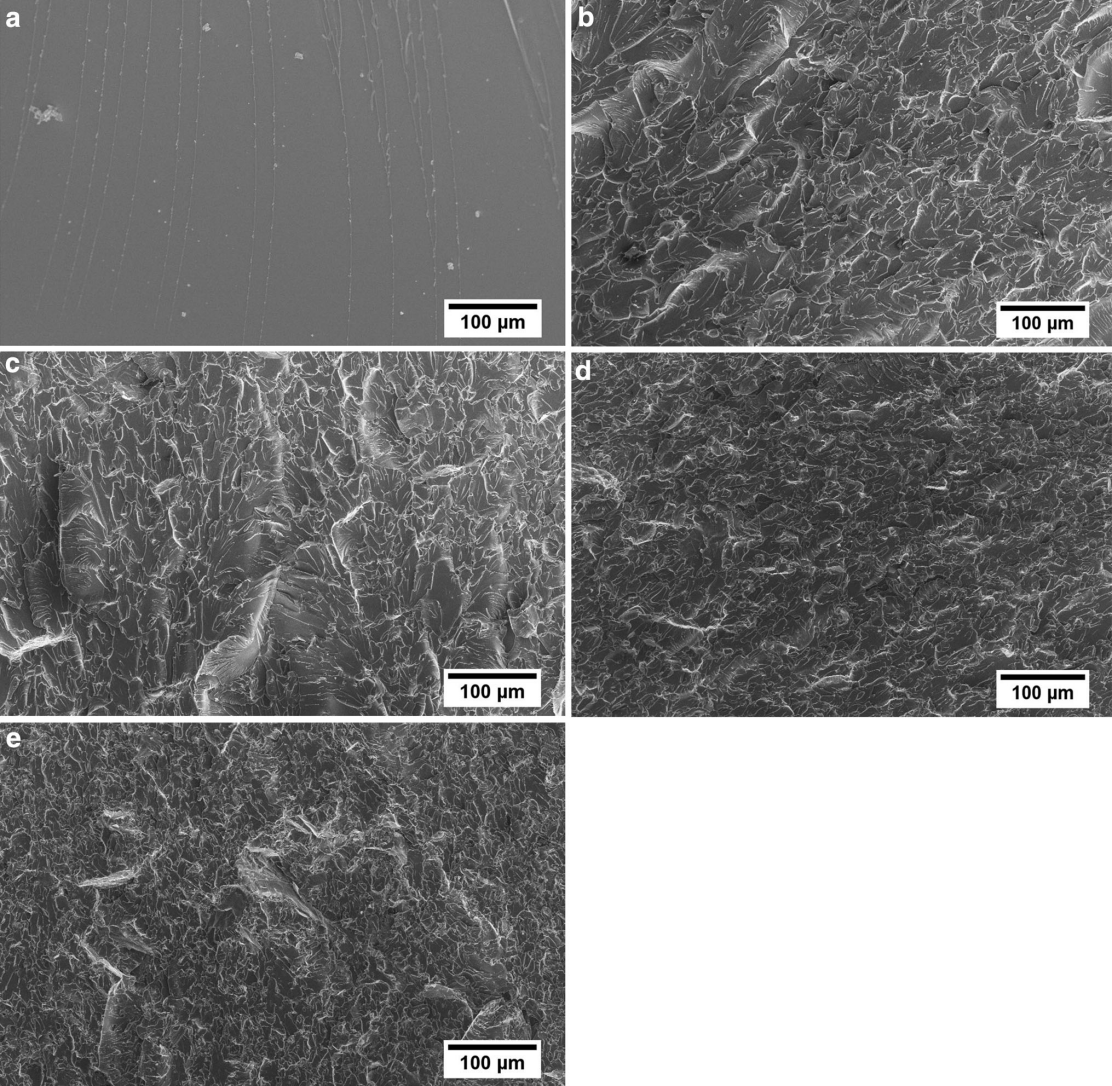
Fracture surface morphology of rGO/epoxy composites examined using FEG-SEM. **a** Neat epoxy, **b** 0.1 wt%, **c** 0.5 wt%, **d** 1 wt%, **e** 2 wt% rGO

The morphologies of the fracture surfaces of the composites are shown in Fig. 2. Although the surface morphology of the neat epoxy (Fig. 2a) appears relatively smooth with river lines characteristic of a brittle fracture, those of the composites (Fig. 2b–e) exhibit increasing surface roughness with increasing rGO content.

It can also be seen in Fig. 2b–e that the rGO appears to be evenly dispersed throughout the composite. This is to be expected due to the fact that the GO contains epoxy groups which would impart good compatibility with the DGEBA resin and improve dispersion during mixing. Furthermore, since significant reduction of the GO takes place at 200 °C, the epoxy matrix would have already gelled at this temperature, therefore preventing re-agglomeration of any unattached rGO during reduction.

### Thermo-mechanical properties

**Fig. 3:**
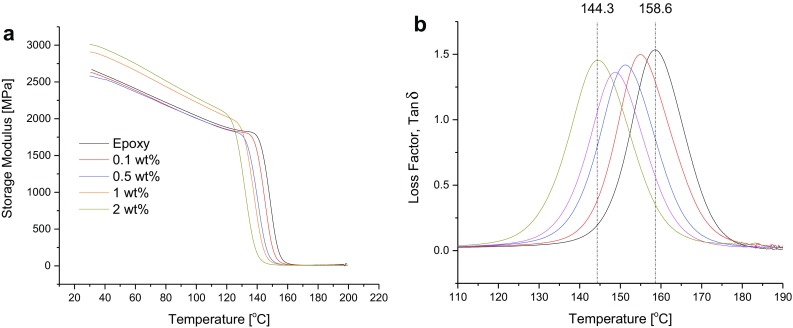
Thermo-mechanical properties of the rGO/epoxy composites

The thermo-mechanical properties of the in situ thermally reduced rGO/epoxy composites are shown in Fig. 3.

From Fig. 3a, the glassy storage moduli, *E*_g_, of the composites, measured at 35 °C, can be seen to increase with increasing rGO content; rising from 2640 MPa for the neat epoxy polymer to 2990 MPa in the epoxy composite filled with 2 wt% rGO (see Table 1). The values of the glass transition temperature, *T*_g_, of the epoxy matrices, which has been taken as the maximum of the tan*δ* curve (Fig. 3b), were however observed to decrease with increasing rGO content from 158.6 °C for the neat epoxy to 144.3 °C for the composite with 2 wt% rGO (i.e. a decrease of nearly 15 °C). As discussed elsewhere (Yang et al. 2009), the increase in the storage modulus may be attributed to good interfacial adhesion between the rGO and the epoxy matrix due to the chemical interaction between the functional groups on the GO and epoxy during the in situ reduction. This chemical interaction between the GO and epoxy may also lead to an increase in the molecular weight between cross-links of the cured epoxy matrix (Zaman et al. 2011; Ma et al. 2013; Yousefi et al. 2013) as the rGO content is increased. The number average molecular weight between cross-links, *M*_nc_, are listed in Table 1. The storage moduli, *E*_r_, in the rubbery region (at 180 °C), from which the *M*_nc_ were calculated according to Eq. (1), are also listed in Table 1. *M*_nc_ increases from 285 g/mol in the neat epoxy to 728 g/mol in the composite filled with 2 wt% rGO. Chen et al. (2013) obtained a similar value of *M*_nc_ for the neat epoxy. The reaction between pendant OCFGs on the GO surface and the epoxy matrix in the liquid phase is well known. Yang et al. (2009) has shown that DGEBA molecules can indeed be grafted onto GO by reaction with pendant OCFGs in an aqueous medium if a mixture of GO and resin is heated at 50 °C while stirring for 4 h. Wan et al. (2014) prepared DGEBA-functionalized GO sheets by dispersing GO sheets in acetone via bath sonication in the presence of DGEBA resin at 70 °C using NaOH as catalyst. They confirmed grafting of DGEBA onto the GO sheets using a combination of XPS, XRD, atomic force microscopy (AFM), transmission electron microscopy (TEM) and Raman spectroscopy. The degassing step adopted in this work for the removal of trapped air (i.e. using 60 °C, −1000 mbar and time of 15 min) may also encourage the reaction of the OCFGs on the GO with the functional groups on the DGEBA resin. For these reasons, the *M*_nc_ increases and the glass transition temperature decreases, as the rGO concentration increases. Addition of GO has also been reported to change the molecular weight of polyurethane (Kim et al. 2010). Nanoclays are also known to have a similar effect on epoxy polymers (Liu et al. 2005; Marouf et al. 2008).

**Table 1:** Thermo-mechanical properties of the rGO/epoxy composites

rGO content (wt%)	Storage modulus, *E*_g_, at 35 °C (MPa)	Storage modulus, *E*_r_, at 180 °C (MPa)	Average molecular weight between cross-links, *M*_nc_ (g/mol)	Glass transition temperature, *T*_g_ (°C)
0.0	2640	11.5	285	158.6
0.1	2690	10.2	321	154.8
0.5	2758	8.4	390	151.2
1.0	2891	6.1	537	148.8
2.0	2990	4.5	728	144.3

### Thermal stability in air

**Fig. 4:**
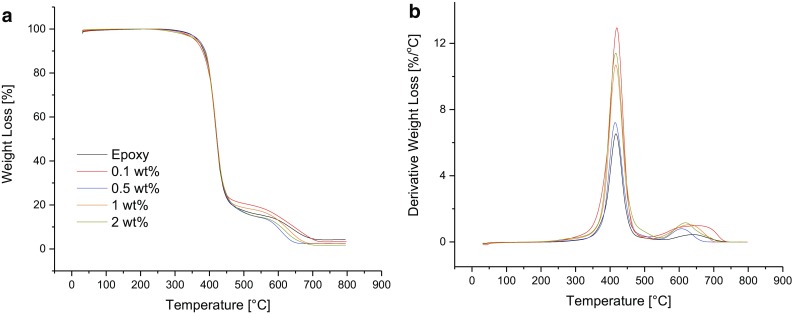
Thermogravimetric analysis of the rGO/epoxy composites

Figure 4 shows the thermal stability of the rGO/epoxy composites in air as measured using TGA. The thermal stabilities of the composites appear to decrease somewhat with increasing rGO content. This can be seen more clearly in Fig. 4b where the rate of thermal degradation of the composites at around 400 °C increases with increasing rGO content. Since the GO is only partially reduced in the composite, the oxygen content of the cured composite may therefore be expected to increase with increasing rGO content. This will lead to an increase in the rate of thermal degradation, as confirmed in Fig. 4b.

### Tensile properties

**Fig. 5:**
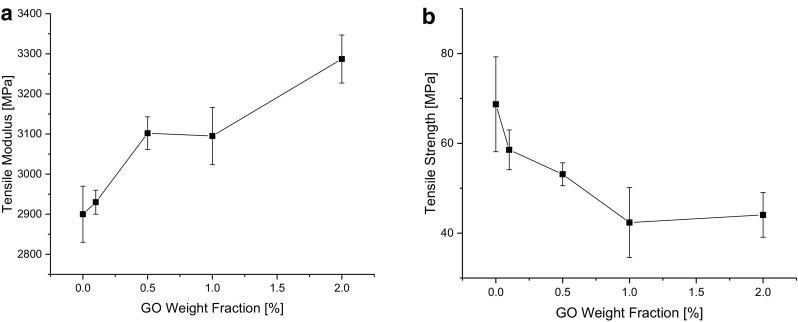
Mechanical properties of the rGO/epoxy composites: **a** Young’s modulus, **b** tensile strength

Figure 5a shows the tensile moduli of the composites. The moduli increase from 2900 ± 70 MPa for the neat epoxy to 3290 ± 60 MPa for the 2 wt% rGO/epoxy composite (i.e. an increase of 13.4 %). Tschoppe et al. (2015) report an increase in the modulus of 8.5 % for an epoxy composite filled with 1.5 wt% of thermally reduced GO and 11.4 % for that filled with 2 wt% of nitrogen-doped thermally reduced GO, whereas Zaman et al. (2011) observed a modest 7 % increase in modulus on adding 2 wt% of 4,4′-methylene diphenyl diisocyanate (MDI)-functionalized GNP to epoxy. The increase in modulus observed in the present work may be ascribed to the relatively high modulus of the rGO filler and to the good interfacial adhesion between the rGO and the epoxy matrix. Good interfacial adhesion may arise from surface chemistry interactions, as well as from mechanical interlocking of the wavy rGO sheets with the epoxy matrix (Yang et al. 2009). As Fig. 5b shows, the tensile strengths of the composites tend to decrease with increasing rGO content. The tensile strength decreases from 68.7 ± 10.6 MPa in the neat epoxy to 44.1 ± 5.0 MPa in the composite with 2 wt% rGO. Such a decrease has been attributed to defects as well as to the change in molecular weight between cross-links of the epoxy matrix, as mentioned above. Zaman et al. (2011) obtained similar results with MDI-functionalized graphene nanoplatelet (GNP)/epoxy composites.

### Thermal conductivity

**Fig. 6:**
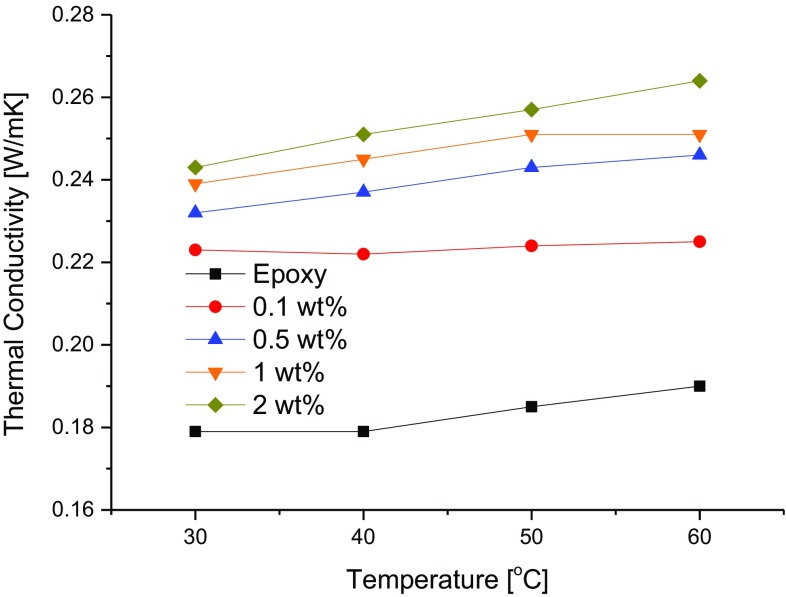
Thermal conductivities of neat epoxy and rGO/epoxy composites at different temperatures

Figure 6 shows the thermal conductivities of the rGO/epoxy composites. The thermal conductivities tend to increase with increasing rGO content and also with increasing temperature, as expected. The maximum thermal conductivity recorded was 0.264 W/mK for the 2 wt% rGO/epoxy composite at 60 °C. The corresponding thermal conductivity of the neat epoxy polymer is 0.190 W/mK. At 30 °C, the corresponding values are 0.243 W/mK and 0.179 W/mK for the 2 wt% rGO/epoxy composite and neat epoxy, respectively (i.e. an increase of about 36 %). The measured thermal conductivities exhibited by these composites are relatively high when compared to the value of 0.21 W/mK reported in the literature for a GNP/epoxy composite (Chandrasekaran et al. 2013). This may be attributed to the excellent dispersion of the rGO in the epoxy resulting from the processing technique adopted in the present work.

## Conclusions

A facile, scalable and commercially viable method has been developed to prepare polymeric composites of a DGEBA epoxy resin with varying rGO content, having improved thermal conductivities. It consists of dispersing freeze-dried GO in a DGEBA epoxy resin using a three-roll mill and subsequently curing at a relatively high temperature with an anhydride curing agent, thereby both partially reducing the GO to rGO in situ and curing the epoxy matrix. The rGO/epoxy composites so produced exhibit a good dispersion of rGO, as observed by electron microscopy, which ensures significant improvements in the thermal conductivity, storage modulus and tensile modulus. Other properties are however slightly reduced, such as the thermal stability, glass transition temperature and tensile strength, probably due to oxygen groups remaining on the embedded rGO and an increase in the molecular weight between cross-links for the epoxy matrices. These results show that it is possible to tune the properties of an epoxy polymer with a simple and viable method of GO addition.
